# Energy spectrum theory of incommensurate systems

**DOI:** 10.1093/nsr/nwae083

**Published:** 2024-03-05

**Authors:** Zhe He, Xin-Yu Guo, Zhen Ma, Jin-Hua Gao

**Affiliations:** School of Physics and Wuhan National High Magnetic Field Center, Huazhong University of Science and Technology, Wuhan 430074, China; School of Physics and Wuhan National High Magnetic Field Center, Huazhong University of Science and Technology, Wuhan 430074, China; School of Physics and Wuhan National High Magnetic Field Center, Huazhong University of Science and Technology, Wuhan 430074, China; School of Physics and Wuhan National High Magnetic Field Center, Huazhong University of Science and Technology, Wuhan 430074, China

**Keywords:** incommensurate system, quasiperiodic potential, plane-wave method, moiré quasicrystal

## Abstract

Because of the lack of translational symmetry, calculating the energy spectrum of an incommensurate system has always been a theoretical challenge. Here, we propose a natural approach to generalize energy band theory to incommensurate systems without reliance on the commensurate approximation, thus providing a comprehensive energy spectrum theory of incommensurate systems. Except for a truncation-dependent weighting factor, the formulae of this theory are formally almost identical to that of Bloch electrons, making it particularly suitable for complex incommensurate structures. To illustrate the application of this theory, we give three typical examples: one-dimensional bichromatic and trichromatic incommensurate potential models, as well as a moiré quasicrystal. Our theory establishes a fundamental framework for understanding incommensurate systems.

## Teaser

This work proposes a natural approach to generalize the energy band theory of Bloch electrons into the incommensurate systems without reliance on the commensurate approximation (supercell), thus providing a comprehensive energy spectrum theory of the incommensurate systems.

## INTRODUCTION

Incommensurate (or quasiperiodic) systems refer to the quantum waves existing in multiple periodic potentials with incommensurate periods. The celebrated examples include the Aubry–André–Harper (AAH) model [1,2], moiré heterostructures [3–18], such as twisted bilayer graphene [3–6], as well as certain types of quasicrystal [19–25]. Distinct from Bloch electrons, incommensurate periodic potentials can lead to many unique phenomena, e.g. wave-function localization [26–36], moiré flat bands and superconductivity [3,37–65], and quasicrystal electronic states with special rotational symmetries forbidden in periodic lattices [66–71], thereby attracting great research interest.

With the rapid technological advancement in recent years, an increasing number of exotic incommensurate systems have been proposed and fabricated in experiments [18,46,72–89], which pose significant challenges to theory. The primary difficulty is that, due to the lack of overall translation symmetry, the Bloch theorem becomes invalid for incommensurate systems. For complex incommensurate structures especially, e.g. twisted trilayer graphene with two arbitrary twist angles [18,75], even the commonly used commensurate approximation is no longer applicable. Therefore, a comprehensive energy spectrum theory for incommensurate systems, which avoids reliance on the commensurate approximation, is urgently required [75,90–93].

In this work, we propose a natural approach to generalize energy band theory of Bloch electrons to incommensurate systems, by which the energy spectrum of incommensurate systems can be conveniently calculated without the need for any commensurate approximation. The formulae of such incommensurate energy spectrum (IES) theory are formally almost identical to those of Bloch electrons, with the only difference being a truncation-dependent weighting factor for each eigenstate. Therefore, it provides a unified method for handling both commensurate and incommensurate potentials, making it an ideal choice for multiple periodic potential models. IES theory establishes a fundamental theoretical framework for comprehending incommensurate systems.

## INCOMMENSURATE ENERGY SPECTRUM THEORY

The one-dimensional (1D) bichromatic incommensurate potential (BIP) model is the simplest incommensurate model [26,28–31], and should thus be the best toy model to illustrate IES theory.

The Hamiltonian of the BIP model is


(1) \begin{eqnarray*}
H = -\frac{\hbar ^2}{2m} \nabla ^2 \!+\! \frac{V_1}{2} \cos (G_1 x) + \frac{V_2}{2} \cos (G_2 x + \phi ),
\end{eqnarray*}

where *V*_1,2_ are the amplitudes of two periodic potentials and *G*_1,2_ are the magnitudes of the reciprocal lattice vectors. We define α = *G*_2_/*G*_1_ as the ratio between the two periods, which is an irrational number in the incommensurate case.

Using the plane-wave basis, we get the Schrödinger equation in momentum space:


(2) \begin{eqnarray*}
&&\frac{\hbar ^2 q^2}{2m} c_q + \frac{V_1}{4} c_{q-G_1} + \frac{V_1}{4} c_{q+G_1} \\
&&+\, \frac{V_2}{4} e^{i\phi } c_{q-G_2} + \frac{V_2}{4} e^{-i\phi } c_{q+G_2} = \varepsilon c_q.
\end{eqnarray*}

Here, *c_q_* is the coefficient of the plane wave with φ(*x*) = ∑_*q*_*c_q_e*^*iqx*^ and ε is the eigenvalue to be determined. Equation (2) represents a set of algebra equations, which couple only the plane-wave states in the set


(3) \begin{eqnarray*}
Q_{q}=\lbrace k\! \mid\! k=q+m G_1+nG_2 :m,n\in \mathbb {Z} \rbrace .
\end{eqnarray*}

Note that, for a given *q*, Equation (2) actually defines a Hamiltonian matrix *H*(*q*) (see the online supplementary material for the definition of *H*(*q*)). To obtain the energy spectrum, some kind of truncation of (*m, n*) should be given first, so that Hamiltonian *H*(*q*) becomes a matrix of finite dimensions. Then, for a given *q*, we can directly calculate the eigenstates of the Hamiltonian matrix *H*(*q*). Now, the key question becomes how to determine a range of values for *q* such that the entire energy spectra of the incommensurate system can be obtained without any omissions or duplications.

To answer this question, let us first review the case of Bloch electrons, i.e. *V*_2_ = 0. In this case, we can also get a set of coupled equations as Equation (2) that are exactly the so-called central equations of the Bloch electrons [94]. Here, the coupled plane waves are $Q^B_{q}=\lbrace k \mid k=q+m G_1:m\in \mathbb {Z} \rbrace$ now. In principle, *q* ∈ (− ∞, +∞). But, to calculate the eigenstates of *H*(*q*), some momenta *q* are equivalent or duplicated. This is because two momenta *q* and *q* + *mG*_1_ ($m \in \mathbb {Z}$) actually correspond to the same set of central equations. In other words, *H*(*q*) and *H*(*q* + *mG*_1_) are the same matrix, thereby yielding identical energy spectra. In this sense, we say that all the momenta in $Q^B_q$ are equivalent. From such equivalence relations, we get two important facts.

1. For any arbitrary momentum *q*, there exists an integer *m*_0_ that yields an equivalent momentum *q*_0_ = *q* + *m*_0_*G*_1_ within the range [0, *G*_1_). This implies that the complete energy spectra can be obtained by considering only momenta within [0, *G*_1_).
2. Any two momenta in the interval [0, *G*_1_) are unequivalent. The implication is that the energy spectra are not computed repetitively, while *q* traverses the interval [0, *G*_1_).

Thus, the conclusion is that, by traversing the interval [0, *G*_1_) with *q*, we can calculate the eigenstates of all *H*(*q*) and obtain the complete energy spectra of the periodic system without repetition. Obviously, this is exactly the standard procedure of energy band theory of Bloch electrons, and the interval [0, *G*_1_) is just the first Brillouin zone (FBZ).

Similar equivalence relations also exist in the incommensurate case. As indicated in Equations (2) and (3), all the momenta in $Q_{q}=\lbrace k \mid k=q+m G_1+nG_2 :m,n\in \mathbb {Z} \rbrace$ are equivalent. Such an equivalence relation can also give us several important messages.

1. With a specified *n*, we can always find an equivalent momentum *q_n_* = (*q* + *nG*_2_) + *m_n_G*_1_ within the range [0, *G*_1_) for any given *q*, where *m_n_* is an integer.
2. Suppose that a truncation of *n* is provided, and that *N_E_* represents the total number of all the allowed *n*. Then, for any *q*, there are *N_E_* equivalent momenta in the interval [0, *G*_1_), which do not overlap with each other due to the incommensurability.

**Figure 1.**
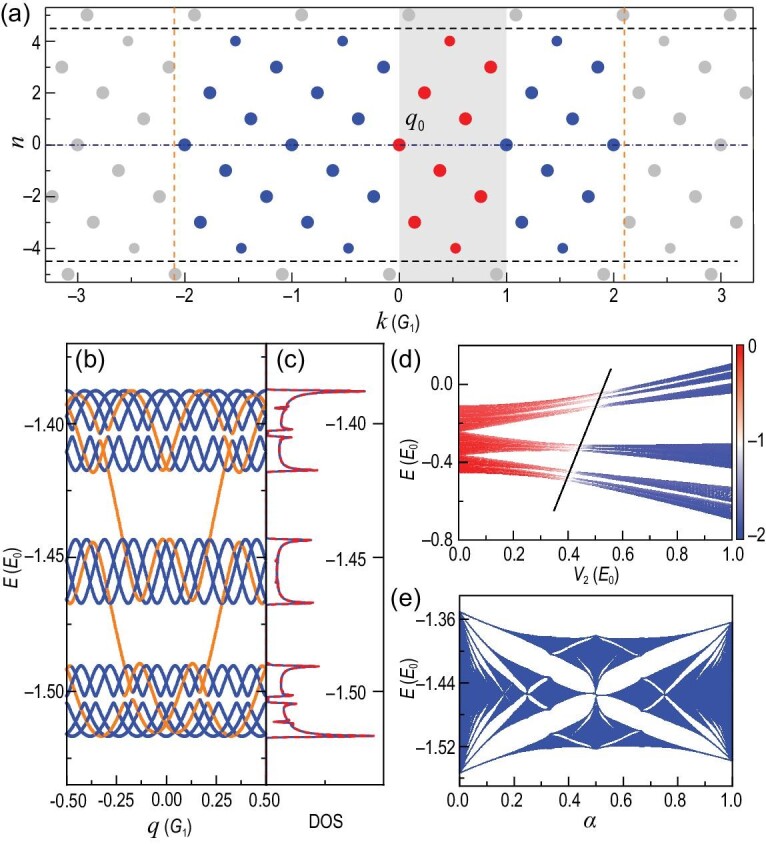
The BIP model with $\alpha =(\sqrt{5}-1)/2$. (a) Schematic of the equivalent momenta for a given *q*. We set *q* = 0 here. (b and c) Energy spectrum diagram and DOS. Orange lines in (b) denote the momentum edge states. Blue lines in (c) represent the DOS obtained from the IES method, while red lines are from the commensurate approximation α ≈ 55/89. Here we set *V*_1_ = 8*E*_0_, *V*_2_ = 0.06*E*_0_ and *n_c_* = 8. (d) IPRM for all the eigenstates. Colors represent log_10_IPRM. Here we set *V*_1_ = 4*E*_0_ and *n_c_* = 40. (e) Butterfly-like spectrum. Here we set *V*_1_ = 8*E*_0_, *V*_2_ = 0.08*E*_0_ and *n_c_* = 12. The other parameters are ϕ = 0, *k_c_* = 4*G*_1_ and *E*_0_ = ($\hbar $^2^/2*m*)(*G*_1_/2)^2^, the energy unit.

Figure 1(a) can help us intuitively understand the two points above. The vertical (horizontal) axis of Fig. 1(a) denotes the value of *n* (*k*), so that all equivalent momenta *k* = *q* + *mG*_1_ + *nG*_2_ for a given *q* can be represented as discrete dots on the graph. Moreover, for a specified *n*, the equivalent momenta (*q* + *nG*_2_) + *mG*_1_ with different values of *m* lie on a horizontal line. Within each horizontal line, we see that there always exists one equivalent momentum within the range [0, *G*_1_) (red dot), which is just the *q_n_* mentioned above. A natural truncation scheme of (*m, n*) is (i) |*n*| ≤ *n_c_*; (ii) |*k*| ≤ *k_c_*. Here, *n_c_* and *k_c_* are two truncation constants, where *k_c_* reflects the truncated energy of the plane waves and *n_c_* determines the minimum interval between all the coupled momenta (see the online supplementary material for the interpretation of the meaning of *n_c_*).

The truncation of *n* (*k*) is represented as the two black horizontal (orange vertical) dashed lines in Fig. 1(a). Once *n_c_* is given, the total number of allowed *n* (red dots) are *N_E_* = 2*n_c_* + 1. It can be proven that all the red dots (*q_n_*) never overlap with each other in the incommensurate case (see the online supplementary material for the proof of this statement). Therefore, for any *q*, we can always find *N_E_* distinct equivalent momenta in the interval [0, *G*_1_).

The two points above suggest that a correct and convenient way to calculate the energy spectra of an incommensurate system is to let *q* traverse the interval [0, *G*_1_), calculate the eigenstates of each *H*(*q*) and assign a weight factor of 1/*N_E_* to each eigenstate.

Based on this idea, the density of states (DOS) and expectation value of an observable in an incommensurate system can be calculated using


(4) \begin{eqnarray*}
\rho (\varepsilon ) = \displaystyle\frac{1}{N_E} \sum _{q \in [0,G_1),i} \delta (\varepsilon - \varepsilon _{qi}),
\end{eqnarray*}

(5) \begin{eqnarray*}
\langle \hat{A} \rangle = \displaystyle\frac{1}{N_E} \sum _{q \in [0,G_1),i} p_{qi} \langle \phi _{qi} |\hat{A}| \phi _{qi} \rangle ,
\end{eqnarray*}

where ε_*qi*_ and |ϕ_*qi*_〉 denote the *i*th eigenvalue and eigenstate of *H*(*q*), and *p_qi_* is the Boltzmann factor. Here, we call the interval [0, *G*_1_) the primary Brillouin zone (PBZ) of incommensurate systems. Note that we can also choose *G*_2_ to be the PBZ, which will give the same results with proper truncation (see the online supplementary material for the calculation results with *G*_2_ as the PBZ). The two formulae above are formally almost identical to that of Bloch electrons, which are the central results of the proposed IES theory.

The IES method can provide accurate enough numerical results as long as the cutoffs *k_c_* and *n_c_* are large enough, since the plane-wave basis is used. In practical calculations, we need to test different truncation values to ensure the convergence of results. An interesting case is *n_c_* → ∞, where the equivalent momenta *q_n_* of an incommensurate model will uniformly and densely fill the whole PBZ. This implies that, once *n_c_* is sufficiently large, the entire incommensurate spectra can be obtained by just calculating the eigenstates of *H*(*q*) with any chosen *q* [75,93]. The cost is that the dimension of the Hamiltonian matrix becomes extremely huge.

The IES method, i.e. Equations (4) and (5), is also valid for the commensurate case. The only difference is that the *N_E_* for the commensurate case has to be determined by directly counting the distinct *q_n_* in the PBZ, since the equivalent momenta in the PBZ (*q_n_*) may now overlap with one another (see the online supplementary material for an example in the commensurate case). In this sense, the incommensurate and commensurate systems exhibit no fundamental distinction, and can therefore be treated under a unified theoretical framework.

## RESULTS

### Results of the bip model

We then apply this IES method to three specific examples for demonstration. The first one is the BIP model, where we set $\alpha =(\sqrt{5}-1)/2$. This is a typical incommensurate case and has been intensively studied with various methods [26,28–31].

The numerical results are given in Fig. 1. First of all, like the Bloch electrons, we can plot energy spectra ε_*qi*_ as a function of *q* [see Fig. 1(b)], where *i* becomes the ‘band’ index and *q* ∈ PBZ. Such an energy spectrum diagram clearly indicates the existence of energy gaps. However, the incommensurate energy spectrum diagram here is different from the energy bands of Bloch electrons, because its shape depends on the *N_E_*, i.e. the truncation *n_c_*. Note that, in the PBZ, there are *N_E_* identical spectra at the *N_E_* equivalent momenta, which is quite unlike that of Bloch electrons. The other obvious characteristic is that there exists a kind of momentum edge state (orange lines) in the energy gaps. Formally, Equation (2) can be viewed as a tight-binding (TB) model in momentum space, where a plane wave is equivalent to a discrete site in momentum space. Thus, when the truncation *n_c_* is given, it actually results in open boundaries in momentum space. Like the TB model in real space, the open boundary will give rise to edge states, i.e. the so-called momentum edge states in Fig. 1(b). Note that, in principle, the truncation of *k_c_* will also give rise to an open boundary, but these boundary states only appear in the high-energy range, since *k_c_* should correspond to the maximum energy of the plane waves. Therefore, for the energy region that we are interested in, only the *n_c_*-induced momentum edge states have to be considered. Since the momentum edge states rely on the truncation, we think that they are artificially induced states that need to be eliminated. We can distinguish the momentum edge states by examining the distribution of the wave functions in momentum space (see the online supplementary material for details about momentum edge states).

To demonstrate the correctness of the IES method, we compare it with the commensurate approximation. The densities of states calculated with the two methods are plotted in Fig. 1(c), where the blue (red) lines represent the IES method (commensurate approximation with α ≈ 55/89). Both methods yield the same DOS, which clearly indicates that the IES method can correctly describe the energy spectrum of the incommensurate system.

The IES method can accurately capture the wave-function characteristics of the incommensurate system as well. A direct example is the single-particle mobility edge of the BIP model, which refers to the quantum localization of the wave functions in an incommensurate system. With the IES method, the inverse participation ratio in momentum space (IPRM) can be utilized to quantify the degree of localization [96],


(6) \begin{eqnarray*}
\mathrm{IPRM} (|\phi _{qi}\rangle ) =\sum _{m,n} |c_{q+mG_1+nG_2}|^4.
\end{eqnarray*}

If the wave function is localized in real space, it should be extended in momentum space, and thus the IPRM will approach zero, i.e. log_10_(IPRM) → −∞, as *k_c_* increases. Conversely, for an extended wave function, log_10_(IPRM) tends to be zero. Figure 1(d) plots the IPRM of all the eigenstates of the BIP model as a function of *V*_2_, where log_10_(IPRM) is represented by color. Clearly, when *V*_2_ is small (large), the wave function is extended (localized). Moreover, we can see that the localization transition is energy dependent (the black oblique line), which indicates that the single-particle mobility edge indeed exists. The results from the IES method are completely consistent with known conclusions [29,30].

Very interestingly, the IES method offers an improved approach to calculate the famous Hofstadter butterfly spectrum. It is known that in the TB limit (with deep *V*_1_ potential), the BIP model will asymptotically approach the AAH model, which can be exactly mapped into a 2D Hofstadter model, i.e. 2D square lattice in a perpendicular magnetic field [97]. Thus, when the energy spectrum of the BIP model is plotted as a function of α, we indeed get a butterfly-like spectrum, as shown in Fig. 1(e). Since α is proportional to the magnetic flux ϕ through each plaquette, this means that IES theory provides a way to calculate the Hofstadter butterfly spectrum under any magnetic field, no matter whether ϕ is rational or irrational.

### Results of the trichromatic incommensurate potential model

The IES method offers a convenient approach for calculating complex incommensurate systems. One example is the 1D trichromatic incommensurate potential (TIP) model, where three applied incommensurate potentials greatly hinder the commensurate approximation. The Hamiltonian is now


(7) \begin{eqnarray*}
H = -\frac{\hbar ^2}{2m} \nabla ^2 + \sum _{i=1,2,3} \frac{V_i}{2} \cos (G_i x),
\end{eqnarray*}

where α_2_ = *G*_2_/*G*_1_ and α_3_ = *G*_3_/*G*_1_ are two irrational numbers [95]. The coupled momenta are now $Q_{q}=\lbrace k \mid k=q+m G_1+n_2G_2 + n_3 G_3:m,n_2,n_3\in \mathbb {Z} \rbrace$. With IES theory, we can still choose [0, *G*_1_) as the PBZ and use the truncation scheme |*n*_2, 3_| ≤ *n_c_* and |*k*| ≤ *k_c_*. Similarly, we also have *N_E_* equivalent momenta in the PBZ, which can be determined by directly enumerating the distinct equivalent momenta in the PBZ. Then, the energy spectrum and other properties can be calculated using Equations (4) and (5) in the same way.

**Figure 2.**
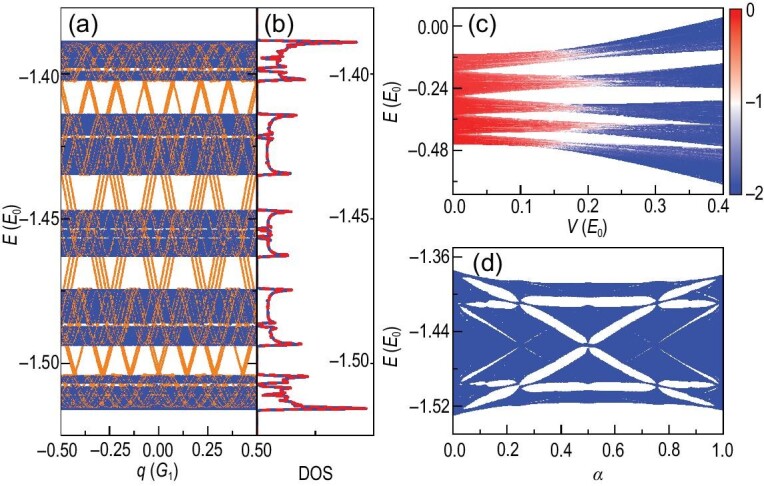
The TIP model. (a and b) Energy spectrum diagram and DOS. Orange lines in (a) denote the momentum edge states. Blue lines in (b) represent the DOS obtained from the IES method, while red lines are from the commensurate approximation *G*_1_: *G*_2_: *G*_3_ ≈ 65: 49: 37. Here we set *V*_1_ = 8*E*_0_, *V*_2_ = *V*_3_ = 0.03*E*_0_ and *n_c_* = 6. (c) IPRM for all the eigenstates. Colors represent log_10_IPRM and we set *V*_2_ = *V*_3_ = *V, V*_1_ = 4*E*_0_ and *n_c_* = 8. (d) Butterfly-like spectrum. Here we set *V*_1_ = 8*E*_0_, *V*_2_ = *V*_3_ = 0.03*E*_0_, *n_c_* = 8, α_2_ = λ^−1^ and α_3_ = α. The other parameters are ϕ = 0, *k_c_* = 4*G*_1_ and *E*_0_ = ($\hbar $^2^/2*m*)(*G*_1_/2)^2^, the energy unit. The two irrational numbers are α_2_ = λ^−1^ and α_3_ = λ^−2^, where λ = 1.3247⋅⋅⋅ is the root of the equation *x*^3^ − *x* − 1 = 0 [95].

The numerical results are given in Fig. 2. Figure 2(a) shows the energy spectrum. With three incommensurate potentials, the number of equivalent momenta *N_E_* is markedly greater than that in the BIP model, resulting in a highly dense energy spectrum. The presence of momentum edge states is also observed in this case (orange lines). We have calculated the corresponding densities of states (blue lines) in Fig. 2(b), which coincide well with those of an approximate commensurate structure (red lines). In Fig. 2(c), we present the IPRM to show the localization feature of the eigenstates. We can also get a butterfly-like spectrum by plotting the energy spectra as a function of α_3_ with a fixed α_2_; see Fig. 2(d).

### Results of the moiré quasicrystal

Two-dimensional incommensurate systems have drawn great interest very recently. Here, we use the IES method to calculate a special 2D incommensurate system, i.e. the moiré quasicrystal, which has eight-fold rotational symmetry but no translation symmetry. Very similar to the BIP model, the moiré quasicrystal can be achieved by applying two square periodic potentials *V*(θ_1_ = 0) and *V*(θ_2_ = π/4) with a twist angle θ = π/4, where


**Figure 3.**
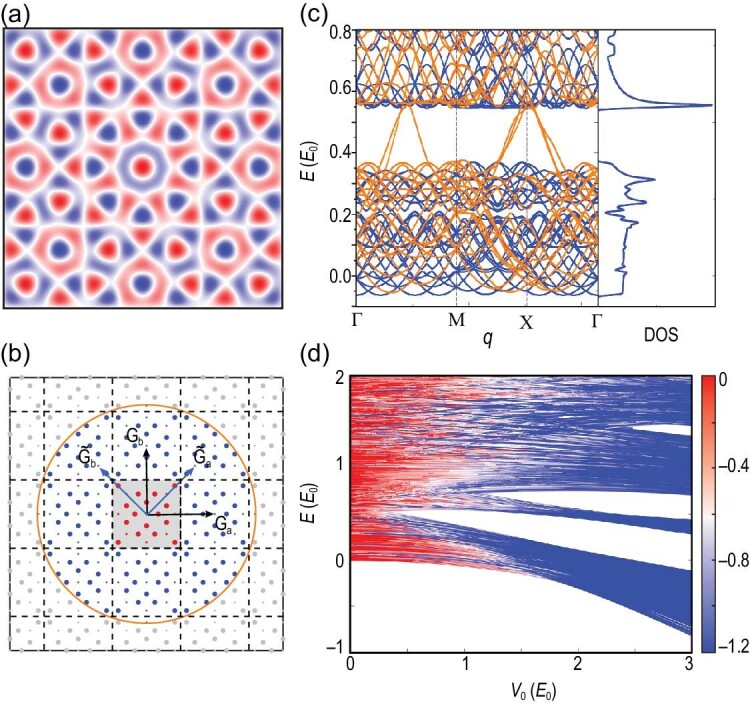
Moiré quasicrystal. (a) Potential of the moiré quasicrystal. (b) Schematic of the equivalent momenta. (c) Energy spectrum and the corresponding DOS. Here we set *V*_0_ = 1*E*_0_, *n_c_* = 4 and *k_c_* = 2.1|**G**_*a*_|. (d) IPRM, where the color represents log_10_(IPRM). Here we set *n_c_* = 7 and *k_c_* = 2.1|**G**_*a*_|. The energy unit *E*_0_ = 2($\hbar $^2^/2*m*)(|**G**_*a*_|/2)^2^.

(8) \begin{eqnarray*}
V(\theta )=\frac{V_0}{4} \lbrace \cos [R(\theta )\mathbf {G}_a\cdot \boldsymbol {r}] + \cos [R(\theta )\mathbf {G}_b\cdot \boldsymbol {r}]\rbrace
\end{eqnarray*}

with **G**_*a*_ and **G**_*b*_ two reciprocal lattice vectors of a square lattice, and *R*(θ) the rotation matrix. The moiré quasicrystal potential is plotted in Fig. 3(a), revealing its evident eight-fold rotational symmetry. Note that the moiré quasicrystal has already been realized in cold-atom systems [23] (see the online supplementary material for the relation to the experimental model).

For a given $\boldsymbol {q}$, the coupled momenta are now $\mathbf {k}=\mathbf {q}+m_1 \mathbf {G}_a+m_2 \mathbf {G}_b + n_1\tilde{\mathbf {G}}_a + n_2\tilde{\mathbf {G}}_b$, where we define $\tilde{\mathbf {G}}_{a,b} \equiv R(\pi /4) \mathbf {G}_{a,b}$ and $m_{1,2}, n_{1,2} \in \mathbb {Z}$. The equivalent momenta and the truncation are illustrated in Fig. 3(b). Here, we choose the FBZ of *V*(θ_1_ = 0) as the PBZ; see the gray square. All the equivalent momenta are plotted as dots in Fig. 3(b), with the truncation scheme |*n*_1, 2_| ≤ *n_c_*, |*k*| ≤ *k_c_*. We denote by *k_c_* the energy cutoff of the plane waves, illustrated as the orange circle. For the moiré quasicrystal, all the equivalent momenta in the PBZ will never overlap with one another (red dots; see the online supplementary material for a proof of this statement), which implies that *N_E_* = (2*n_c_* + 1)^2^. The calculated DOS is given in Fig. 3(c). In Fig. 3(d), we plot the IPRM of the moiré quasicrystal. IES theory does provide a convenient method for handling the moiré quasicrystal. All the calculation details of the three examples are given in the online supplementary material.

## SUMMARY

More generally, besides the energy spectrum calculation above, our theory actually offers a convenient way to transplant all the formulae of energy band theory into incommensurate systems. Thus, it in fact provides a fundamental framework for comprehending incommensurate systems.

## Supplementary Material

nwae083_Supplemental_File
