# Supplementary material for: Energy spectrum theory of incommensurate systems
Source: Natl Sci Rev. 2024 Mar 5;11(12):nwae083. doi: 10.1093/nsr/nwae083 (PMC11660950; doi:10.1093/nsr/nwae083)
Supplement: nwae083_Supplemental_File [file nwae083_supplemental_file.pdf]

Supplementary Materials for: Energy Spectrum Theory of Incommensurate Systems

Zhe He,[1] Xin-Yu Guo,[1] Zhen Ma,[1] and Jin-Hua Gao[1,*]

[1]*School of Physics and Wuhan National High Magnetic Field Center, Huazhong University of Science and Technology, Wuhan 430074, China*

I. THE MATRIX REPRESENTATION OF HAMILTONIAN

In the main text, we get the Schrödinger equation in momentum space using a plane wave basis, i.e. Eq. (2). For a chosen value of q, we can arrange all the coupled plane waves in a certain order, e.g., $(\cdots, q-G_1, q, q+G_1, q+2G_1, \cdots, q+G_2, q+G_2+G_1, q+G_2+2G_1, \cdots)$. Then, Eq. (2) actually define a Hamiltonian matrix $H(q)$ as

$$H(q) = \begin{pmatrix} \ddots & & & & & & & & & \\ & \varepsilon_{q-G_1} & \frac{1}{4}V_1 & & & & & & & \\ & \frac{1}{4}V_1 & \varepsilon_q & \frac{1}{4}V_1 & & \cdots & \frac{1}{4}V_2e^{-i\phi} & & & \\ & & \frac{1}{4}V_1 & \varepsilon_{q+G_1} & \frac{1}{4}V_1 & & & \frac{1}{4}V_2e^{-i\phi} & & \\ & & & \frac{1}{4}V_1 & \varepsilon_{q+2G_1} & & & & \frac{1}{4}V_2e^{-i\phi} & \\ & & \vdots & & & \ddots & & & \vdots & \\ & & \frac{1}{4}V_2e^{i\phi} & & & & \varepsilon_{q+G_2} & \frac{1}{4}V_1 & & \\ & & & \frac{1}{4}V_2e^{i\phi} & & & \frac{1}{4}V_1 & \varepsilon_{q+G_2+G_1} & \frac{1}{4}V_1 & \\ & & & & \frac{1}{4}V_2e^{i\phi} & \cdots & & \frac{1}{4}V_1 & \varepsilon_{q+G_2+2G_1} & \\ & & & & & & & & & \ddots \end{pmatrix}, \tag{1}$$

where $\varepsilon_q = \dfrac{\hbar^2 q^2}{2m}$.

II. THE EQUIVALENT MOMENTA IN PBZ

For the BIP model, the incommensurate central equations indicate that all the momenta in the set $Q_q = \{k | k = q + mG_1 + nG_2 : m, n \in \mathbb{Z}\}$ are all equivalent. The equivalent relations imply a fact: for a given q, there are N_E equivalent momenta in the PBZ, which do not overlap with each other in the incommensurate case. Here, we give a proof of this statement.

N_E is the total number of the all the allowed n, and each n corresponds to an equivalent moment $(q + nG_2) + mG_1$ in PBZ, where m is an integer. Suppose that q_n and $q_{n'}$ are two equivalent momenta in PBZ with $n \neq n'$, i.e.

$$\begin{aligned} q_n &= (q + nG_2) + m_n G_1 \\ q_{n'} &= (q + n'G_2) + m_{n'} G_1 \end{aligned} \tag{2}$$

where n, n', m_n ane $m_{n'}$ are integers. If the two momenta coincide, we get

$$q + nG_2 + m_n G_1 = q + n'G_2 + m_{n'} G_1 \tag{3}$$

which means

$$\frac{G_1}{G_2} = \frac{m_{n'} - m_n}{n - n'} = \frac{1}{\alpha}. \tag{4}$$

α now becomes a rational number, which is clearly in contrast to the incommensurate assumption. So, the conclusion is that all the N_E equivalent momenta in PBZ will never overlap with one another.

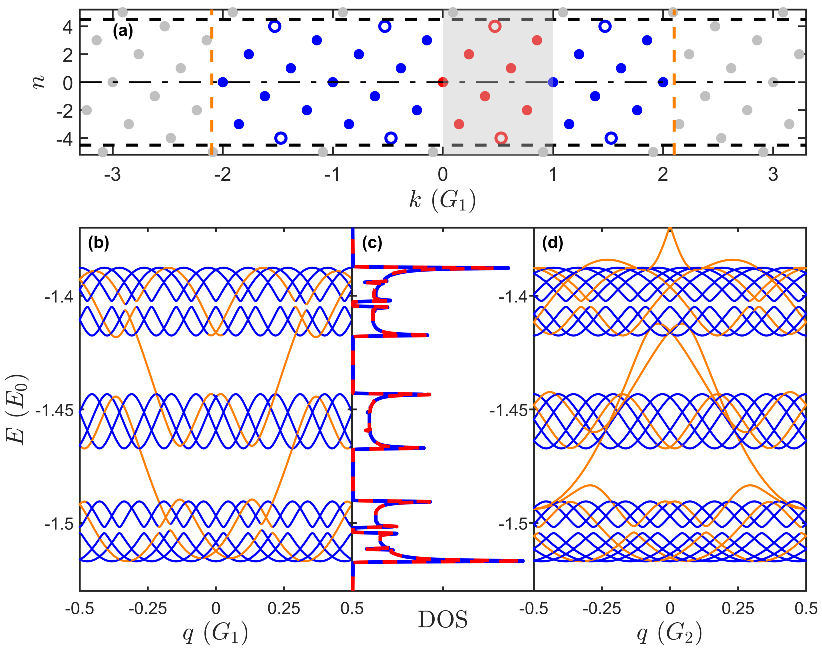

FIG. S1: (a) Schematic of the momentum edge states in the BIP model with $\alpha = (\sqrt{5}-1)/2$. The n_c induced momentum edge states are represents as the hollow dots. (b) is same as the Fig. 1 (b) of the main text. Parameters: $V_1 = 8E_0$, $V_2 = 0.06E_0$, $n_c = 8$, $\phi = 0$, $k_c = 4G_1$. (d) is the results with G_2 as the PBZ. Parameters: $V_1 = 8E_0$, $V_2 = 0.06E_0$, $n_c = 10$, $\phi = 0$, $k_c = 4G_1$. (c) DOS, blue lines represent (b), and red lines represent (d).

This statement can be generalized to the 2D incommensurate systems. Here, we use the 2D moire quasicrystal as an example. For a given $\mathbf{q}$, all the equivalent momenta can be expressed as $\mathbf{q} + m_1\mathbf{G_a} + m_2\mathbf{G_b} + n_1\tilde{\mathbf{G}}_\mathbf{a} + n_2\tilde{\mathbf{G}}_\mathbf{b}$. Suppose that $\mathbf{q_n}$ and $\mathbf{q_{n'}}$ are two equivalent momenta in PBZ with $(n_1, n_2) \neq (n_1', n_2')$. If they coincide, we have

$$\mathbf{q} + m_1\mathbf{G_a} + m_2\mathbf{G_b} + n_1\tilde{\mathbf{G}}_\mathbf{a} + n_2\tilde{\mathbf{G}}_\mathbf{b} = \mathbf{q} + m_1'\mathbf{G_a} + m_2'\mathbf{G_b} + n_1'\tilde{\mathbf{G}}_\mathbf{a} + n_2'\tilde{\mathbf{G}}_\mathbf{b} \tag{5}$$

It means

$$(m_1 - m_1')\mathbf{G_a} + (m_2 - m_2')\mathbf{G_b} = (n_1' - n_1)\tilde{\mathbf{G}}_\mathbf{a} + (n_2' - n_2)\tilde{\mathbf{G}}_\mathbf{b} \tag{6}$$

where right side (left) side is a lattice vector of the first (second) periodic potential V_1 (V_2), i.e. $(m_1 - m_1')\mathbf{G_a} + (m_2 - m_2')\mathbf{G_b}$ is a common lattice vector for both V_1 and V_2. Note that both V_1 and V_2 are square periodic potentials, which has C_4 symmetry. Therefore, we know that $(m_1 - m_1')R(\pi/4)\mathbf{G_a} + (m_2 - m_2')R(\pi/4)\mathbf{G_b}$ is a common lattice vector for both V_1 and V_2 as well. Such two common lattice vectors in momentum space actually corresponds to a supercell of the moire quasicrystal, which is in contrast to the incommensurate assumption. So, we get the conclusion that all the equivalent momenta will never overlap with each other.

III. MOMENTUM EDGE STATES

As explained in the main text, the truncation of the n_c will give rise to momentum edge states mostly in the energy gaps, due to appearance of the open boundary in momentum space. It is also mentioned that the truncation of k_c will also give rise to open boundary, but these boundary states only appear in the high energy range, because that k_c should correspond to the maximum energy of the plane waves. Therefore, for the energy region that we are interested in, only the n_c induced momentum edge states has to be considered. In other words, the momentum edge states in Fig. 1 (b) of the main text are mainly distributed around the n_c induced boundaries, which are illustrated in Fig. S1 (a) (hollow dots).

Because that the momentum edge states depend on the truncation, they are artificially induced states, which should be eliminated in the calculations. In practice, we identify the momentum edge states by examining the wave function distribution in the momentum space. The eigenstates will be deleted from the final results, if they distribute mainly around the boundaries. Note that, the boundary is not just a line, but has width. So, we define n_e as the boundary

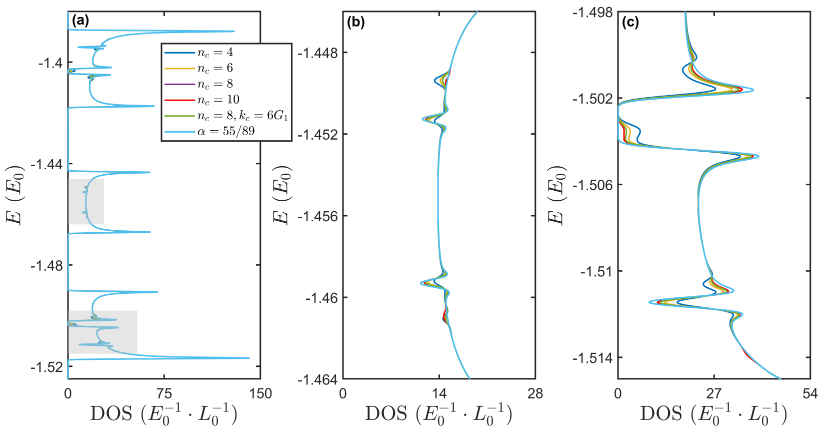

FIG. S2: The DOS of the BIP model with different n_c and k_c. (b) and (c) are the enlarged DOS plots for the gray regions in (a). Parameters: $V_1 = 8E_0, V_2 = 0.06E_0, \alpha = (\sqrt{5}-1)/2, k_c = 4G_1$.

width, which means the outermost n_e sites are viewed as the boundary regions. The N_E should be corrected when some equivalent momenta are near the boundary, i.e. $N_E = 2(n_c - n_e) + 1$.

Of course, The criterion of the boundary states is not unique, which can be further improved in subsequent works. However, because that the bulk states actually dominate in such systems, small changes of the boundary criteria will not affect the final results.

IV. THE PHYSICAL SIGNIFICANCE OF THE TRUNCATION

To calculate the incommensurate energy spectrum, we need to introduce a truncation of (m, n) to get a finite-dimensional Hamiltonian matrix $H(q)$. As discussed in the BIP model, the used truncation is $|n| < n_c$ and $|k| < k_c$, where n_c and k_c are two provided truncation constants. The physical meaning of k_c is very clear, which represents the cutoff of the energy of plane waves. Meanwhile, n_c also has a clear physical significance, which indeed reflects the minimal interval between the equivalent momenta for given q. Actually, due to the incommensurability, the combination of G_1 and G_2, i.e. $mG_1 + nG_2$, can give rise to arbitrarily small momentum, which is essentially different from the commensurate case. When $|n| < n_c$, no matter what the value of m is, $|mG_1 + nG_2|$ always has a minimum value, which depends on the value of n_c. Such physical pictures of the truncation is valid for all the incommensurate systems.

V. PRIMARY BRILLOUIN ZONE

In principle, it is better to use a deep potential as the PBZ, and a shallow potential as a perturbation. The advantage lies in the fact that shallow perturbation can lead to a small truncation n_c, as demonstrated in the main text. The converse is also valid, but the truncation should be larger. In Fig. S1 (c) and (d), we use the interval $[0, G_2)$ as the PBZ and calculate the DOS and energy spectrum. We see that, with a larger truncation n_c, we get the same DOS.

VI. THE BIP MODEL

Here, we give some calculation details of the BIP model. Fig. S2 plots the calculated DOS with different truncation n_c and k_c, which illustrates the convergence of the results. The DOS results show that we can get a convergent numerical results even with truncation $n_c = 4$. And the results from the IES theory are in good agreement with the commensurate approximation as well. To calculate the DOS, we uniformly choose 1000 q points in the PBZ. For each q point, the $H(q)$ is a 137×137 matrix with $n_c = 8$ and $k_c = 4G_1$.

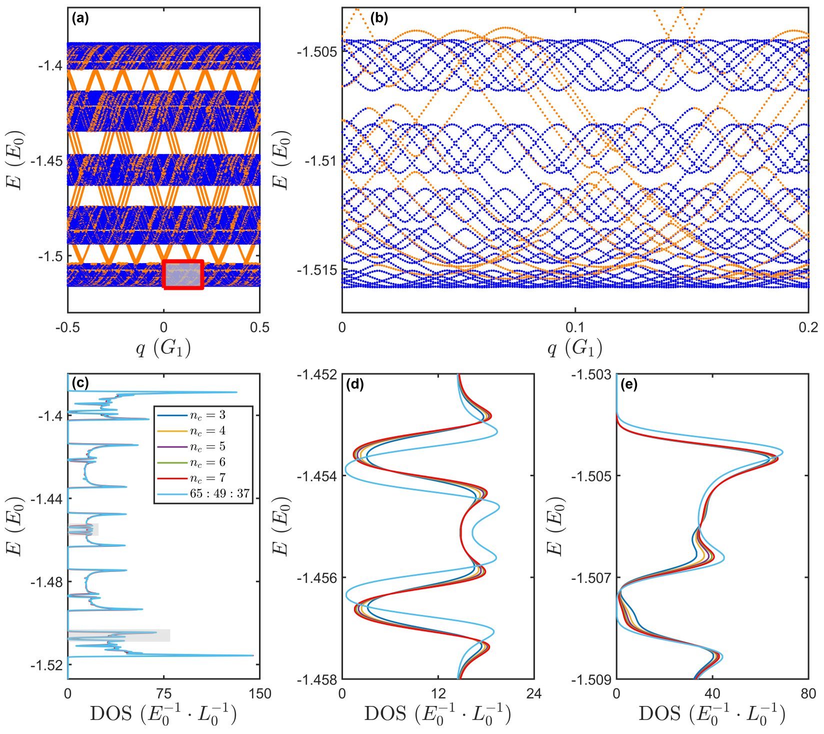

FIG. S3: The TIP model. (a) is the energy spectrum diagram, the same as the Fig. 2 of the main text. (b) and (c) are the enlarged energy spectrum plot for the region of the red box in (a). (c) is the DOS calculated with different truncation n_c, and the results of a commensurate approximation is also given. (d) and (e) are enlarged DOS plots for the gray regions in (c). Parameters: $V_1 = 8E_0, V_2 = V_3 = 0.03E_0, k_c = 4G_1$. The two irrational numbers are $\alpha_2 = \lambda^{-1}$, $\alpha_3 = \lambda^{-2}$, where $\lambda = 1.3247\cdots$ is the root of the equation $x^3 - x - 1 = 0$ [1].

VII. THE TIP MODEL

Here, we give some calculation details of the TIP model. Fig. S3 (a) is the same as the Fig. 2 of the main text. Then, we plot the enlarged energy spectrum diagram (region in the red box) in Fig. S3 (b). The DOS with various truncations are shown in Fig. S3 (c) to illustrate the numerical convergence, where Fig. S3 (c) and (d) are the enlarged DOS plots in the gray regions. The DOS of a commensurate approximation ($G_1 : G_2 : G_3 \approx 65 : 49 : 37$) is also given as a comparison . First, we see that a convergent DOS can be achieved even with $n_c = 3$. Meanwhile, Fig. S3 (d) and (e) indicate that the IES theory may give better results than that of the common commensurate approximation. It is because that, with the IES theory, the predicted position of peaks always remains unchanged with increasing n_c. In Fig. S3, $H(q)$ is a 393×393 matrix when $n_c = 3$.

VIII. MOIRE QUASICRYSTAL

First, we would like to show that the moire quasicrystal model here is exactly the same as that in Ref. 23 of the main text. In Ref. 23, the potential is

$$V(r) = V_0 \sum_{i=1}^{D=4} \cos^2\left(\frac{\mathbf{G_i}}{2} \cdot r\right) \tag{7}$$

$$= \frac{V_0}{2} \sum_{i=1}^{D=4} \cos(\mathbf{G_i} \cdot r) + 2V_0 \tag{8}$$

If we set $\mathbf{G_1} = \mathbf{G_a}$, $\mathbf{G_3} = \mathbf{G_b}$, $\mathbf{G_2} = \tilde{\mathbf{G}}_\mathbf{a}$ and $\mathbf{G_4} = \tilde{\mathbf{G}}_\mathbf{b}$, we then obtain the moire quasicrystal potential in the main text, except for a factor $\frac{1}{2}$ for V_0 and a constant potential shift.

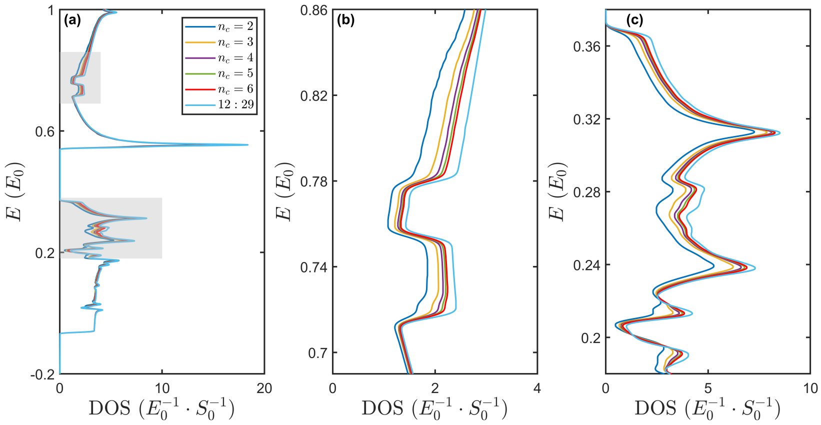

FIG. S4: The DOS of moiré quasicrystal with different n_c. (b) and (c) are the enlarged plots for the gray regions in (a). Parameters: $V_0 = 1E_0, k_c = 2.1|\mathbf{G_a}|$.

In Fig. S4, we plot the calculated DOS with different truncation to illustrate the convergence. The results of a commensurate approximation structure is given as well. Here, a commensurate approximation structure is described by two integers (m, n) with

$$\cos\theta_{mn} = \frac{n^2 - m^2}{m^2 + n^2}, \tag{9}$$

where θ_{mn} is the twisted angle [2]. For the moire quasicrystal, $(m, n) = (12, 29)$ corresponds to $\theta_{mn} \approx 44.96°$, which is a reasonable commensurate approximation. The calculated results indicate that the truncation $n_c = 4$ has already given a satisfied DOS results. The DOS is calculated with a 400×400 mesh in PBZ.

IX. THE COMMENSURATE CASES

Here, we use the bichromatic potential model as an example to illustrate that the IES formulae are also valid for the commensurate case. Specifically, we consider a commensurate situation: $G_1 : G_2 = 3 : 1$. Based on the Eq. (2) of the main text, we can also plot the equivalent momenta for a given q with G_1 as the PBZ, see Fig. S5 (a). Clearly, distinct from the incommensurate case, the equivalent momenta will overlap in such commensurate case. Thus, we eventually get three distinct equivalent momenta in the PBZ, i.e. $N_E = 3$, which is irrelevant to the n_c. Note that $[0, G_1/3)$ is just the FBZ of such commensurate case. Therefore, Eq. (4) and (5) of the main text do give the correct DOS and expectation values. The energy spectrum and DOS are given in Fig. S5 (b). This example clearly demonstrates that the IES theory actually is applicable to the commensurate cases. So, the IES formulae in fact provides a unified theoretical framework to treat the multi periodic potential models, no matter whether it is incommensurate or not. In practical calculations, the only issue is to correctly determine N_E by counting the distinct equivalent momenta in the PBZ. Note that in the commensurate cases, n_c has no effect and k_c is the only meaningful truncation.

* Electronic address: jinhua@hust.edu.cn
[1] Casati G, Guarneri I and Shepelyansky DL. Anderson transition in a one-dimensional system with three incommensurate frequencies. *Phys. Rev. Lett.* 1989; **62**: 345–348.
[2] Wang P, Zheng Y, Chen X *et al.* Localization and delocalization of light in photonic moiré lattices. *Nature* 2020; **577**: 1–5.

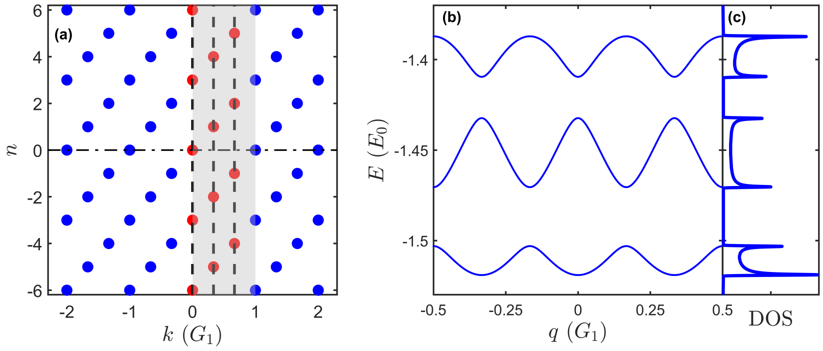

FIG. S5: The commensurate bichromatic potential model with $G_1 : G_2 = 3 : 1$. (a) shows the equivalent momenta. (b) and (c) are the energy spectrum and DOS. Parameters: $k_c = 4G_1, V_1 = 8E_0, V_2 = 0.06E_0, \phi = 0$.
